# Identifying high-affinity aptamer ligands with defined cross-reactivity using high-throughput guided systematic evolution of ligands by exponential enrichment

**DOI:** 10.1093/nar/gkv534

**Published:** 2015-05-24

**Authors:** Agata Levay, Randall Brenneman, Jan Hoinka, David Sant, Marco Cardone, Giorgio Trinchieri, Teresa M. Przytycka, Alexey Berezhnoy

**Affiliations:** 1 Sylvester Comprehensive Cancer Center, University of Miami Miller School of Medicine, Miami, FL 33136, USA; 2 National Center of Biotechnology Information (NCBI), National Library of Medicine (NLM), National Institutes of Health (NIH), Bethesda, MD 20894, USA; 3 Cancer Inflammation Program, Center for Cancer Research, National Cancer Institute (NCI), National Institutes of Health (NIH), Frederick, MD, USA; 4 Department of Medicine, University of Miami Miller School of Medicine, Miami, FL 33136, USA

## Abstract

Oligonucleotide aptamers represent a novel platform for creating ligands with desired specificity, and they offer many potentially significant advantages over monoclonal antibodies in terms of feasibility, cost, and clinical applicability. However, the isolation of high-affinity aptamer ligands from random oligonucleotide pools has been challenging. Although high-throughput sequencing (HTS) promises to significantly facilitate systematic evolution of ligands by exponential enrichment (SELEX) analysis, the enormous datasets generated in the process pose new challenges for identifying those rare, high-affinity aptamers present in a given pool. We show that emulsion PCR preserves library diversity, preventing the loss of rare high-affinity aptamers that are difficult to amplify. We also demonstrate the importance of using reference targets to eliminate binding candidates with reduced specificity. Using a combination of bioinformatics and functional analyses, we show that the rate of amplification is more predictive than prevalence with respect to binding affinity and that the mutational landscape within a cluster of related aptamers can guide the identification of high-affinity aptamer ligands. Finally, we demonstrate the power of this selection process for identifying cross-species aptamers that can bind human receptors and cross-react with their murine orthologs.

## INTRODUCTION

Research and development of targeted inhibitors, such as therapeutic antibodies, recombinant ligands and aptamers, is a rapidly progressing field (1). The problem of how to make safer and more efficient drugs was traditionally approached by increasing the affinity of a ligand for its target, while at the same time reducing non-specific interactions (2,3). The generation of ligands with controllable cross-reactivity is another, arguably superior, alternative. For example, broadly cross-reactive antibodies are able to neutralize polymorphic viruses such as human immunodeficiency virus (HIV) and influenza (4). The cross-reactivity of therapeutic ligands recognizing rodent and non-human primate orthologs would also be highly beneficial for assessing on-target toxicity.

Therapeutic antibodies have a number of disadvantages, including dose-limiting toxicities, long circulation times, and high production costs. Additionally, generating cross-reactive antibodies can be a lengthy and time-consuming process of trial and error (5). An alternative platform of designed ligands, oligonucleotide aptamers, has been shown in preclinical studies to be an equivalent, and sometimes superior, alternative to antibodies (6–10). Like antibodies, these ligands are able to recognize their targets with high specificity and comparable avidity. However, unlike antibodies, aptamers can be produced using cell-free chemical synthesis and are therefore less expensive to manufacture and more amenable to post-production modification. A number of high-profile publications have illustrated the feasibility and therapeutic potential of aptamers as specific inhibitors and targeting ligands for the inhibition of tumor growth and HIV replication (6–10).

Early adoption of aptamers has been plagued by difficult selection procedures and poor affinity to certain targets, typically those with lower isoelectric points (p*I*). The introduction of high-throughput sequencing (HTS) has enabled the identification of aptamers early during selection and has significantly facilitated the aptamer isolation process (11), reducing time and cost and minimizing PCR bias and the loss of high-affinity aptamers due to target competition. Nonetheless, HTS for systematic evolution of ligands by exponential enrichment (HTS-SELEX) in its current form is a challenging protocol, given the massive amount of sequencing data that need to be analyzed. Because HTS-SELEX is essentially a non-competitive process, the prevalence of aptamers in the selected pool is rarely indicative of their binding properties or function. Furthermore, factors such as carrier substrate affinity, weak electrostatic interactions with the target, and, in particular, PCR-generated biases can lead to the expansion of ‘passenger’ sequences and could, in the absence of a highly parallel characterization approach, render the selection useless.

Here, we employ a novel bioinformatics-guided selection and screening strategy to rapidly isolate aptamers against two different targets: the human interleukin (IL)-10 and human 4-1BB receptors.

## MATERIALS AND METHODS

### *In vitro* selection

A DNA template for the selection library was ordered from IDT (Coralville, IA). A total of 1 nmol each of the N40 template (5′-TCTCGATCTCAGCGAGTCGTCGNNNNNNNNNNNNNNNNNNNNNNNNNNNNNNNNNNNNNNNNCCCATCCCTCTTCCTCTCTCCC-3′) and the 5′ primer (5′-GGGGGAATTCTAATACGACTCACTATAGGGAGAGAGGAAGAGGGATGGG-3′) were annealed together, extended with Taq polymerase (Life Sciences, Thermo Fisher Scientific; Lafayette, CO, USA), and transcribed *in vitro* using the Durascribe T7 *In Vitro* Transcription (IVT) kit (Illumina, San Diego, CA, USA). The random R0 RNA was purified by denaturing 10% polyacrylamide gel electrophoresis (PAGE), precleared with human IgG-coated (Sigma-Aldrich Corp. St. Louis, MO, USA) Protein A Sepharose beads (GE Healthcare, Little Chalfont, Buckinghamshire, United Kingdom), and then used for *in vitro* selection. In total, 1 nmol R0 RNA was co-incubated with 0.3 nM bead-bound human IL-10RA-Fc fusion protein (Novus Biologicals, CO, USA) in 100 mM NaCl selection buffer (20 mM HEPES, 2 mM CaCl_2_, 0.1 mg/ml BSA). After several washes, the recovered bound RNA fraction was reverse-transcribed using an AMV RT kit (Life Sciences, Thermo Fisher Scientific; Lafayette, CO, USA) and the reverse primer (5′-TCTCGATCTCAGCGAGTCGTCG-3′). cDNA was amplified by either ePCR or oPCR using a Platinum Taq PCR kit (Life Sciences, Thermo Fisher Scientific; Lafayette, CO, USA), as described below. These DNA templates were then used to generate the IVT RNA for the next round of selection. During subsequent rounds, the amount of target protein was reduced by 25% at each step, and the concentration of NaCl was gradually increased to 150 mM. cDNA was amplified using a Platinum Taq PCR kit with the addition of 10% PCRx enhancer solution and the following primers: 5′-GGGGGAATTCTAATACGACTCACTATAGGGAGAGAGGAAGAGGGATGGG-3′ and 5′-TCTCGATCTCAGCGAGTCGTCG-3′. After preparing the master mix PCR reaction solution, it was separated into 100-μl aliquots, and each aliquot was mixed with 600 μL of ice-cold oil fraction assembled from components supplied in the emulsion PCR kit (EuRx Ltd, Gdansk, Poland), according to the manufacturer's instructions. This mixture of oil and water was emulsified by vortexing (3000 rpm) at +4°C and amplified using standard PCR conditions (94°C for 30 s, 55°C for 30 s, and 72°C for 30 s) for 25 cycles. For the selection of aptamers against the human 4-1BB protein, we used the DNA template oligo (5′-TCTCGATCTCAGCGAGTCGTCGNNNNNNNNNNNNNNNNNNNNNNNNNNNNNNNNNNNNNNNNCCCATCCCTCTTCCTCTCTCCC-3′) and the respective 5′ (5′-GGGGGAATTCTAATACGACTCACTATAGGGAGAGAGGAAGAGGGATGGG-3′) and 3′ (5′-TCTCGATCTCAGCGAGTCGTCG-3′) primers to prepare and amplify the DNA libraries between selection rounds.

### High-throughput sequencing and data processing

RNA pools representing different steps in the *in vitro* selection process were reverse transcribed using cloned AMV RT enzyme and a set of barcoded reverse primers (CACCACACC-barcode-CAAAACTAG). Corresponding cDNA products were amplified by ePCR with ACACAC-barcode-AGAC forward and CACCACACC reverse primers. The products were gel purified and amplified using ePCR with PE1.0 and PE2.0 Illumina primers. The resulting indexed and adapter-fused libraries were pooled and submitted for NGS using a HiSeq 2500 device and a 100-cycle paired-end sequencing protocol.

The resulting HTS datafile in a fastq format was uploaded to the NCBI data server and analyzed using HTS aptamotive software, as described elsewhere (12). The prevalence of each individual sequence was identified in every analyzed pool, including at different rounds of selection and in various pools from the same selection round. All identified sequences were clustered using the algorithm described previously (12). Based on copy number and cluster association, sequences were described as either bystander sequences (sequences seen only once in the entire dataset) or true aptamers (present in multiple copies and/or present in different pools). Among the true aptamer sequences, we also distinguished between those belonging to a cluster and those that were unclustered or that had no related sequences in the dataset.

### Normalization of aptamer prevalence in the presence of targets with significantly different p*I* values

The mIL10RA protein has a predicted p*I* value of 8.58 (net positive charge at pH 7.5), whereas hIL-10RA has a p*I* of 6.86 (net negative charge at pH 7.5). We incubated two aliquots of the RNA library with identical amounts of both proteins in normal selection buffer (150 mM NaCl, pH 7.5) and measured the prevalence of aptamers retained by both targets using NGS. To account for p*I* differences, another aliquot of the same RNA library was incubated with a control protein (hIgG, p*I* = 7.2) in the same buffer (pH 7.5) under conditions where the net charge of the target was predicted to be positive (pH 6.8), therefore facilitating non-specific interactions. We found that many aptamers that did not interact with the control protein under normal conditions could efficiently bind to it in the adjusted buffer, causing them to be significantly enriched. These data were used to identify and exclude those sequences influenced by electrostatic interactions (Supplementary Figure S1).

### *In vitro* binding studies

*In vitro* measurements of candidate aptamer affinities to their respective targets were performed using a double filter-binding assay (13). *In vitro* transcribed and PAGE-purified RNAs were dephosphorylated with calf intestinal alkaline phosphatase (CIAP; New England Biolabs, MA, USA) and radiolabeled with polynucleotide kinase (PNK; New England Biolabs MA, USA) using P32 gamma-ATP (Perkin Elmer, MA, USA). Radiolabeled aptamer RNAs were incubated individually with a range of concentrations of target or control proteins at 37°C in binding buffer. Complex formation was determined by passing the mixture through stacked nitrocellulose and NYLon membranes (Whatman, GE healthcare, Little Chalfont, Buckinghamshire, United Kingdom) and then measuring the amount of radioactivity retained on the nitrocellulose (bound RNA fraction) and NYLon (unbound fraction) membranes using a phosphoimager screen (Kodak, NY, USA) and a Typhoon instrument (Amersham Biosciences, GE Healthcare, Little Chalfont, Buckinghamshire, United Kingdom). *K*_d_s were calculated as the concentration of protein required to retain half of the RNA in the RNA:protein complex.

## RESULTS

### Selection of hIL-10RA-binding aptamers begins to converge at round 5

**Figure 1.**
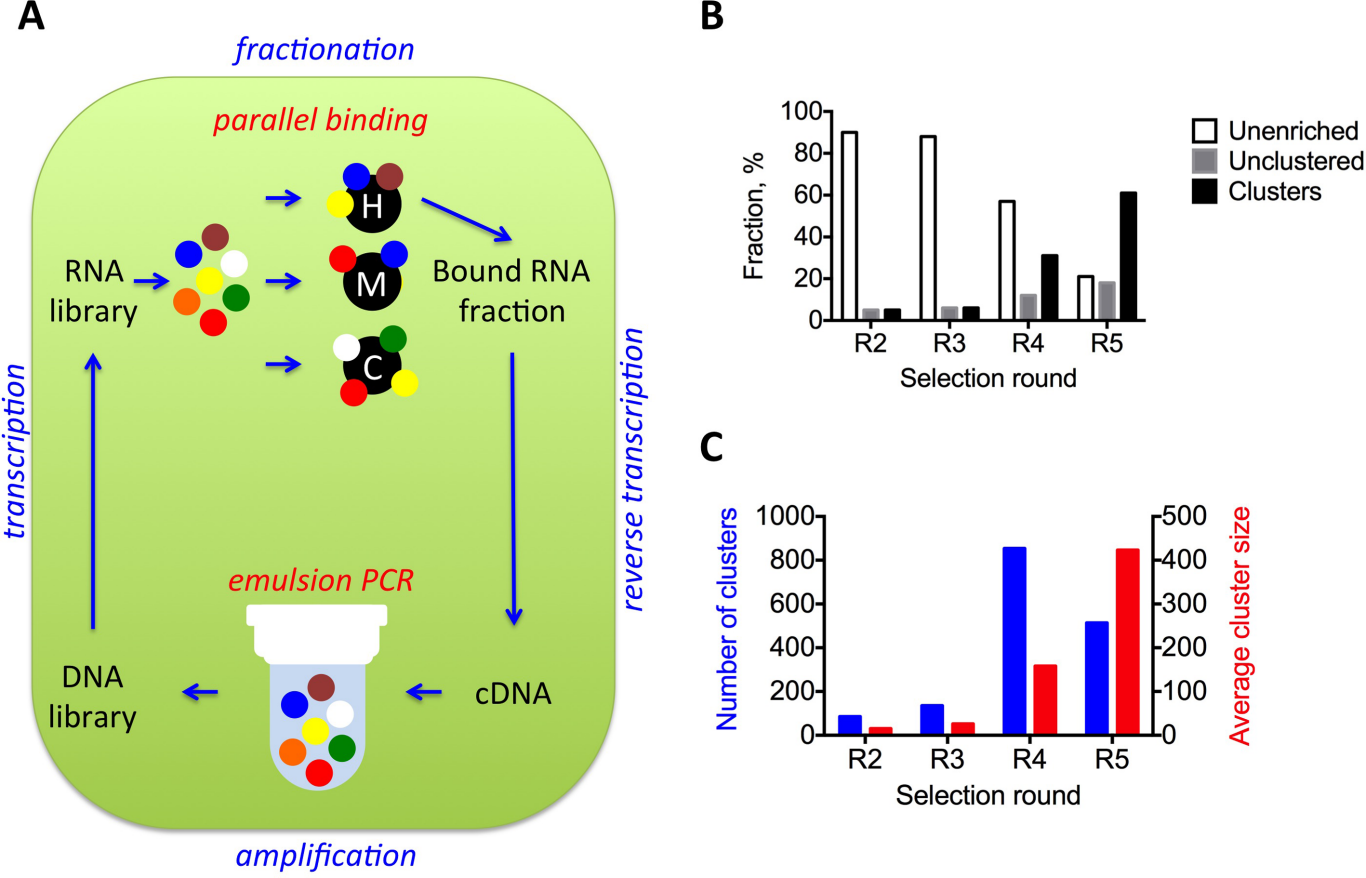
Convergence of the *in vitro* selection begins at round 5. (**A**) Schematic representation of *in vitro* selection process and its key improvements: at amplification step we employed emulsion PCR to preserve variability of the selected library by physical separation of amplification of individual DNA molecules; at fractionation step we introduced parallel binding there identical aliquotes of the same library had been reacted with different targets (depicted in black), for example (H)uman protein, (M)urine ortholog of it and (C)ontrol, to deduce aptamers’ binding patterns in highly parallel format. Bound RNA portions from hIL-10 SELEX rounds 2–5 were isolated, mass sequenced and analyzed for cluster formation. (**B**) Fractions of single copy aptamers (unenriched, open bars), aptamers with multiple copies but did not belonging to any cluster (unclustered, gray bars) and aptamers belonging to a cluster plotted (clustered, black bars). (**C**) Total number of individual clusters (blue bars, left Y axis) and average number of members per cluster (red bars, right Y axis) in each round.

To isolate aptamers recognizing human IL-10RA with high affinity and specificity, we performed *in vitro* selection for five rounds, gradually decreasing the amount of target protein (Figure 1A and Supplementary Table S1). RNA recovered from rounds 2 to 6 was sequenced as described previously (14). On average, 3 × 10^6^ (2 × 10^6^ to 5 × 10^6^) reads were recovered from each pool. After demultiplexing, as described in the Materials and Methods section, we used a hash-based algorithm to determine the prevalence of individual sequences. We define ‘bystander’ sequences as those encountered only once in the dataset in a single copy and ‘true aptamer’ sequences as present in multiple rounds and/or in multiple copies (Table 1). As described previously, we used k-mer distance to cluster related sequences (12). Highly similar sequences with as many as three mismatches/deletions/insertions were associated with the same cluster. The results, including aptamer sequences, their prevalence in each pool, and their cluster association, are hosted online on NCBI servers in a MySQL database format as described previously (15).

**Table 1.** Representative dataset from the selection of aptamers against human IL-10 receptor

Cluster ID	Sequence ID	N40 sequence	Prevalence R4	Prevalence R5	Enrichment rate	Kd of hIL10RA binding, nM
Representative core^a^ sequences from some major clusters						
B	401	CCCCCGCATCACGCCGTGGTGCGATTGACACAATTGCAAT	103 513	421 606	4.1	25
C	402	TCACAGTCCCGGTGCCGCACTAAAACCCATTGTTGTGCGA	37 630	149 129	4.0	120
D	403	TGAGAACTTCTCTCAGTCGGTGGGAGAGTACATCCTAACA	23 423	6081	0.3	>500
K	406	TAGACGAGCACTTCTCTCAGTCGCATTCATTATTTAAATT	16 011	3058	0.2	>500
G	407	CCCCTTCCAGCGATTACGATCATTGACTCTCAGTCCTGTG	11 789	4679	0.4	>500
H	408	ATCGACAGCTCTCAGTCCATTCGAGGAATGTTCATCGATA	5637	1738	0.3	>120
J	411	AGCCATGACGATGTCGTTACGTAGATGCAGAGACTCCTAA	3961	6311	1.6	18
Z	436	TAACACTCGATTCTCCTAGCCCGCTAGAAATTCCCCTCCC	2513	76 374	30.4	65
#3	446	AAAGACCGTTTTTTAAAACGCTCAATATACACGACATAAA	89	409	4.6	10
#25	454	TGAATCTCGCGCTCGTTGGTACCCTTAAAATAAAGGCATA	742	3147	4.2	8
Representative sequences from cluster J*						
J	#411-J	AGCCATGACGATGTCGTTACGTAGATGCAGAGACTCCTAA	3961	6311	1.6	18
J	#411-J-A1G	**G**GCCATGACGATGTCGTTACGTAGATGCAGAGACTCCTAA	14	47	3.4	9
J	#411-J-G7T	AGCCAT**T**ACGATGTCGTTACGTAGATGCAGAGACTCCTAA	14	25	2.6	18
J	#411-J-A19T	AGCCATGACGATGTCGTT**T**CGTAGATGCAGAGACTCCTAA	1	2	2	n.d.**
J	#411-J-C28T	AGCCATGACGATGTCGTTACGTAGATG**T**AGAGACTCCTAA	2	17	8.5	18
J	#411-J-G30T	AGCCATGACGATGTCGTTACGTAGATGCA**T**AGACTCCTAA	9	46	4.9	18
**J**	#411-J-A39G	AGCCATGACGATGTCGTTACGTAGATGCAGAGACTCCT**G**A	4	5	1.2	n.d.
Representative unclustered and bystander sequences						
Unclustered^b^	1BTCIHHiD	TGAAGACTTCTCAGTGCCTCGTCGTACTAAAAACGCAATA	0	3	n.d.	n.d.
	1TJjuBP	GCAGCTATCTCACCGAAAGCGTCGCTATATTCGTCGTTAT	1	3		
Bystander^c^	none	CTATCGCGGCCGATTGTTTCTGCGGACGATGTTGTCCTCA	0	1		
	none	TAGTTTGGGCATGTTTCCCTTGTAGGTGTGAAACCACTTA	0	1		

^a^ Core sequence defined as the most prevalent aptamer of the cluster.

^b^ Unclusteres defined as sequences represented in multiple copies but different from any other aptamer by four or more substitutions/insertions/deletions.

^c^ Bystander defined as sequence appeared only once in selection.

* Differences in sequence between core sequence and variant depicted as **bold** letter.

** Values not determined.

The fraction of bystander sequences decreased over early rounds of the selection (Figure 1B), whereas the number of true aptamers and aptamer clusters and their sizes increased steadily for each round. However, at round 5, the number of unique clusters started to decrease, whereas their average size continued to rise (Figure 1C). We hypothesize that these changes were reflective of a selection process that proceeded from a non-competitive to a competitive stage and eventually converged. As converged selection can lead to the extinction of multiple useful sequences, we decided to stop the process at round 5 and analyze the results.

### Emulsion PCR preserves greater library diversity during selection

The capacity of emulsion PCR (ePCR) to uniformly amplify complex mixtures has been demonstrated to benefit the *in vitro* selection of ribozymes (16) and DNA-based aptamers (17,18). Here, we used this *in vitro* compartmentalization technique to optimize the PCR amplification step of the ‘RNA-based’ hIL-10RA aptamer SELEX protocol. To investigate the effects of ePCR, we amplified cDNA from the second round (R2) using either ePCR or open PCR (oPCR), performed another round of selection (R3), and then used HTS to compare the prevalence of individual sequences in the pools amplified by oPCR or ePCR.

**Figure 2.**
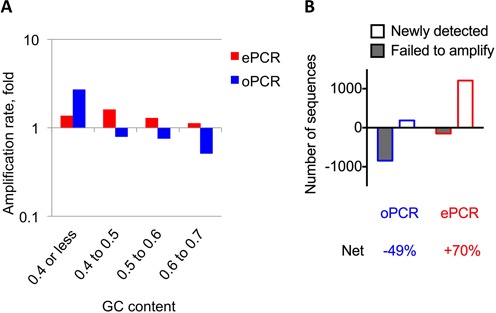
ePCR preserves library diversity better than regular PCR during *in vitro* selection. The cDNA from the second round of hIL-10RA SELEX was amplified by either ePCR or oPCR. After identical transcription, purification and *in vitro* selection procedures, recovered RNA fractions were sequenced, and the recovered pool of individual sequences in both libraries was directly compared. (**A**) Fifty highly enriched sequences from third round of the IL-10RA aptamer selection were pooled based on the GC content of N40 region. The average enrichment rate for each of the pools amplified by ePCR (red bars) or oPCR (blue bars) was determined. (**B**) Analysis of previously detected sequences at round 2 in round 3 shows many more sequences fail to amplify (grey bars) using oPCR (blue outline), whereas more unique sequences (open bars) were detected using ePCR (red outline) as the amplification method. The net result calculated represents the final percent diversity calculation for the resulting library when subtracting previously detected sequences from newly detected ones indicating that ePCR preserves greater library diversity.

Whereas most of the highly enriched sequences were present in both pools, their rates of amplification by the two PCR methods were significantly different depending on the CG content of the random N40 region (Supplementary Table S2). As predicted, ePCR amplified all candidate sequences evenly, whereas oPCR efficiency was inversely correlated with the N40 region GC content (Figure 2A). For example, sequences with the lowest GC content were amplified by oPCR ∼3-fold more efficiently compared with their amplification rate by ePCR, whereas the prevalence of sequences with the highest GC content was significantly reduced in the oPCR-generated pool.

These differences in amplification rate significantly impacted the prevalence of highly enriched aptamers, although most of the candidates were recovered at round 3 after amplification by both methods. Among 1337 of the less abundant sequences (copy number 100 or less) identified at R2, only 492 were recovered using oPCR, indicating that ∼67% of these sequences were lost, thereby reducing library diversity (Figure 2B). By contrast, ePCR recovered most (>88%) of the low-abundance sequences (Figure 2B). Additionally, as selection progressed from R2 to R3, more sequences from the library were amplified to the threshold of HTS detection. Using oPCR, we were able to detect 185 new candidate sequences in the pool, whereas ePCR identified six-fold more (1208).

Overall, library amplification from R2 to R3 by oPCR revealed a significant (49%) loss of sequence diversity, whereas *in vitro* compartmentalization using ePCR prevented this loss, in fact leading to an ∼70% increase in library sequence diversity. Extrapolating these differences to five consecutive rounds of selection underscores the limitations of oPCR and the value of ePCR for maintaining library diversity.

### High-affinity aptamers can be identified by enrichment rate

Preserving and expanding the diversity of the SELEX library between selection rounds is desirable, although it also increases the pool of candidates that need to be analyzed, which can be challenging. Thus, developing effective criteria for identifying high-affinity binding aptamers is becoming paramount. Here, we compared the predictive values of prevalence and enrichment rate for aptamers isolated by ePCR-driven selection (Table 1 and Supplementary Table S3).

**Figure 3.**
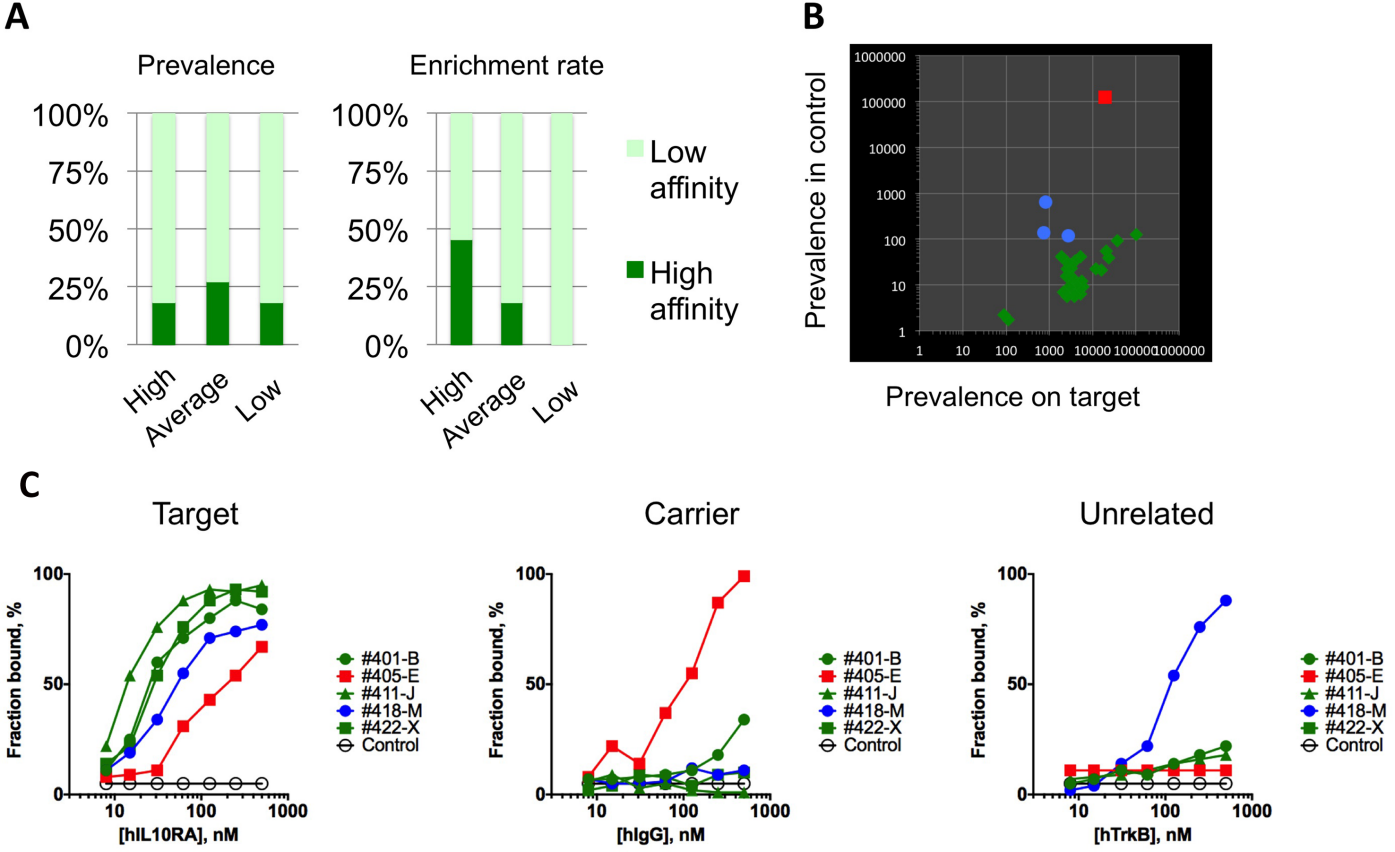
Characterization of aptamers that bind to human IL­10RA with high affinity. (**A**) Aptamers were tested for their capacity to bind hIL­10RA protein in solution using a standard double-filter-binding assay (see Materials and Methods section) and were ranked by either their prevalence (left) or their enrichment rate (right). High-affinity aptamers (*K*_d_ < 20 nM, dark green) and low-affinity aptamers (*K*_d_ > 20 nM, bright green). (**B**) Prevalence of candidate aptamers at round 5 in the pools recovered from hIL10RA (X axis) or hIgG (Y axis) proteins. Aptamers with a ten-fold higher prevalence in the hIL10RA pool than in the hIgG pool (green), aptamers with a ten-fold lower prevalence in the hIL10RA pool than in the hIgG pool (blue), and aptamers that were more prevalent in the hIgG pool (red). (**C**) Binding affinities of representative aptamers to the target (hIL-10RA), nonspecific control (TrkB), or carrier (hIgG) were measured using the double filter-binding assay described in the Materials and Methods section.

Representative sequences from all major clusters of IL10-RA selection were generated by *in vitro* transcription and tested for binding to recombinant hIL-10RA protein. Overall, we identified seven sequences that bound to the protein target with a *K*_d_ value lower than 20 nM (Supplementary Table S3). Ranking the sequences by copy number, we determined that these high-affinity binders were distributed evenly among sequences with high, low and medium prevalence (Figure 3A, left panel). However, the high-affinity aptamers generally showed high enrichment rates (Figure 3A, right panel). A notable exception was sequence 436-Z, which was the aptamer with the highest enrichment rate that also had a medium *K*_d_ for the target (Table 1). To confirm and further investigate this observation, we applied the same analysis to the HTS data from another *in vitro* selection performed against the h4-1BB protein using 20nt random part that allows representing full complexity of the library in the starting pool, and therefore, excluding any bias in the starting material (Supplementary Table S4). We found that, although some of the high-affinity 4-1BB-binding aptamers could not be identified by prevalence at any given round of selection, most of them showed above-average enrichment rates (Supplementary Table S5). Similar to the hIL-10RA aptamers, there were exceptions—two aptamers with the highest enrichment rates also had relatively high *K*_d_s. Interestingly, combination of both enrichment rate and prevalence seems to be the a good indicator of the high affinity binders, as four out of seven hIL-10RA binders ranked high in both parameters.

Overall, our data confirm previous observations that enrichment rate is a better predictor of high-affinity binding aptamers (19) and underscores the importance of analyzing multiple rounds of selection and the need to design post-selection aptamer-screening strategies based on library dynamics.

### Using reference pools to determine aptamer specificity in a parallel format

Enrichment of sequences in SELEX protocols based on factors other than target binding is common for aptamers and antibodies. We hypothesized that parallel selection with a similarly structured but unrelated target and then comparing the prevalence of the candidates between the two selections could be used to distinguish between target-driven and nontarget-driven amplified aptamers. To test this hypothesis, after the third round of selection, we divided the oligonucleotide library into two pools that were then incubated with either the protein of interest (hIL-10RA-Fc fusion) or the control protein (hIgG).

We identified three general distribution patterns: (i) sequences that were enriched preferentially in the hIL-10RA pool; (ii) sequences that were present in both pools in comparable amounts; and (iii) sequences that were enriched preferentially in the control pool (Figure 3B and Supplementary Table S3). Notably, sequence #405-E, which was enriched in the control pool, was among the most prevalent in the IL-10RA pool and would have been considered a useful candidate if not for this comparison.

Next, we tested the affinity of representative sequences from each group to human IgG or to an unrelated hTrkB-hFc fusion protein (Supplementary Table S3). Although we observed no IgG-binding aptamers among the candidates that were preferentially enriched against hIL-10RA, the sequences enriched against IgG showed significant affinity to human IgG (Figure 3C). Interestingly, sequences that were prevalent in both the IgG and the IL-10RA pools demonstrated only modest affinity to both proteins, whereas at least one showed some level of binding to TrkB, reflecting possible non-specific interactions (Figure 3C).

To extend these observations, we applied the same analysis to the selection of aptamers against the human 4-1BB receptor. We found no sequences that were highly represented in the hIgG pool, and consistent with this observation, none of the sequences identified in the course of this selection showed affinity to IgG (Supplementary Table S5). Similar to the hIL10RA selection, incubation with an unrelated target identified a few sequences, such as RJB#294, that were also enriched in the control pool. Subsequent analysis showed that none of these candidates had high affinity to the 4-1BB target, whereas some demonstrated modest binding to the unrelated protein, possibly due to non-specific electrostatic interactions (data not shown).

Overall, we concluded that the deep sequencing of reference pools could help to identify and eliminate candidates with nonspecific binding.

### Mutational landscapes of HTS-SELEX data can identify aptamers with increased affinity

PCR-generated mutagenesis during the SELEX procedure gives rise to new sequences during the selection process (14). In addition to potentially increasing binding affinity, such mutations can reveal important structural information concerning the effects of specific nucleotides on binding affinity. To identify such favorable mutations, we determined the average frequency of such mutations within a cluster of related sequences. The total number of copies of all derivative sequences—presumably derived from a ‘founding’ sequence with the highest prevalence—for each particular cluster was divided by the copy number of the founding sequence and the constant 40, which is the number of nucleotides in the random region. Interestingly, whereas overall cluster size varied greatly, the substitution rate was comparable across many clusters, averaging 0.001–0.005 at each position, yielding 10–50 variants with different substituted positions per 10 000 copies of founding sequence.

**Figure 4.**
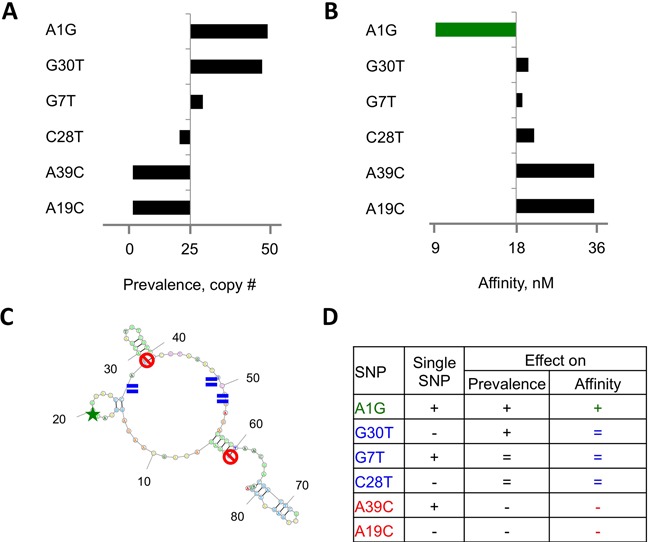
Mutational landscape identifies binding-improving mutations in selected aptamers. (**A**) Prevalence at round 5 of aptamers from cluster #411-J bearing substitutions at positions 1, 7, 19, 28, 30 and 39 had been compared to the expected rate (32). (**B**) Affinity of the same aptamers had been compared to affinity of the cluster founder (18 nM). (**C**) Substituted positions 1, 7, 19, 28, 30 and 39 had been mapped on predicted secondary structure of #411-J aptamer with green color indicating affinity-improving mutation, blue indicating substitutions resulted in aptamer with the same affinity and red indicates binding disrupting mutations. (**D**) Summary of effects nucleotide position on binding function of aptamer #411-J. SNP, substitutions ID; single SNP, type of substitution in this position encountered among cluster forming aptmers, represented by single substitution variant (+), or multiple different variants (−).

Next, we compared the prevalence of every substitution-bearing variant with the average substitution rate for the cluster generated by the #411-J founding sequence (Table 1 and Supplementary Table S6). Whereas the majority of positions were substituted at the average rate, a few variants bearing certain mutations, such as A at position 1 substituted to G (A1G) and G30T, were significantly more abundant (Table 1; Figure 4A). A small number of positions had unusually low numbers of mutations, such as A39 or A19 (Figure 4A). We hypothesized that mutations that increase affinity would be found at positions with higher rates of substitution, as higher affinities would cause these sequences to be preserved during selection. To test this hypothesis, we generated two variants each of the high- (A1G and G30T), average- (G7T and C28T), and low-frequency (A11C and A39C) mutations and measured their respective affinities to the hIL-10RA protein relative to the founding sequence.

As expected, sequences with an average substitution rate showed unchanged binding to the target, whereas the two low-frequency mutations showed reduced binding, and one of the two high-frequency mutations, A1G, exhibited higher affinity (Figure 4B). Mapping these mutations to the predicted secondary structure (Figure 4C) of the #411-J aptamer did not provide a suitable explanation as to why one but not the other mutation caused an increase in affinity. In fact, the only difference we could identify was that position 1 featured only one particular substitution, A1G, whereas position 30 showed all three possible variants (G30T, G30A and G30C) present in the pool at the average rate (Figure 4D, Table 1 and Supplementary Table S6). Although mechanistic studies will need to be performed to understand the relationship between nucleotide position and binding affinity, our results indicate that substitution pattern analysis can be used to guide the identification of aptamers with superior affinity.

### Cross-species reactive aptamers are enriched in both human and murine IL10RA-bound pools

To test whether we could identify a human IL-10RA-binding aptamer that also bound to the murine ortholog of this protein, the library was divided into two parts and subjected to a round of selection against either human or murine IL-10RA targets. Bound fractions were subjected to HTS and prevalence analysis, as described above. We also adjusted the binding conditions to exclude sequences that bound proteins via nonspecific electrostatic interactions (Supplementary Figure S1, see Materials and Methods section for details).

**Figure 5.**
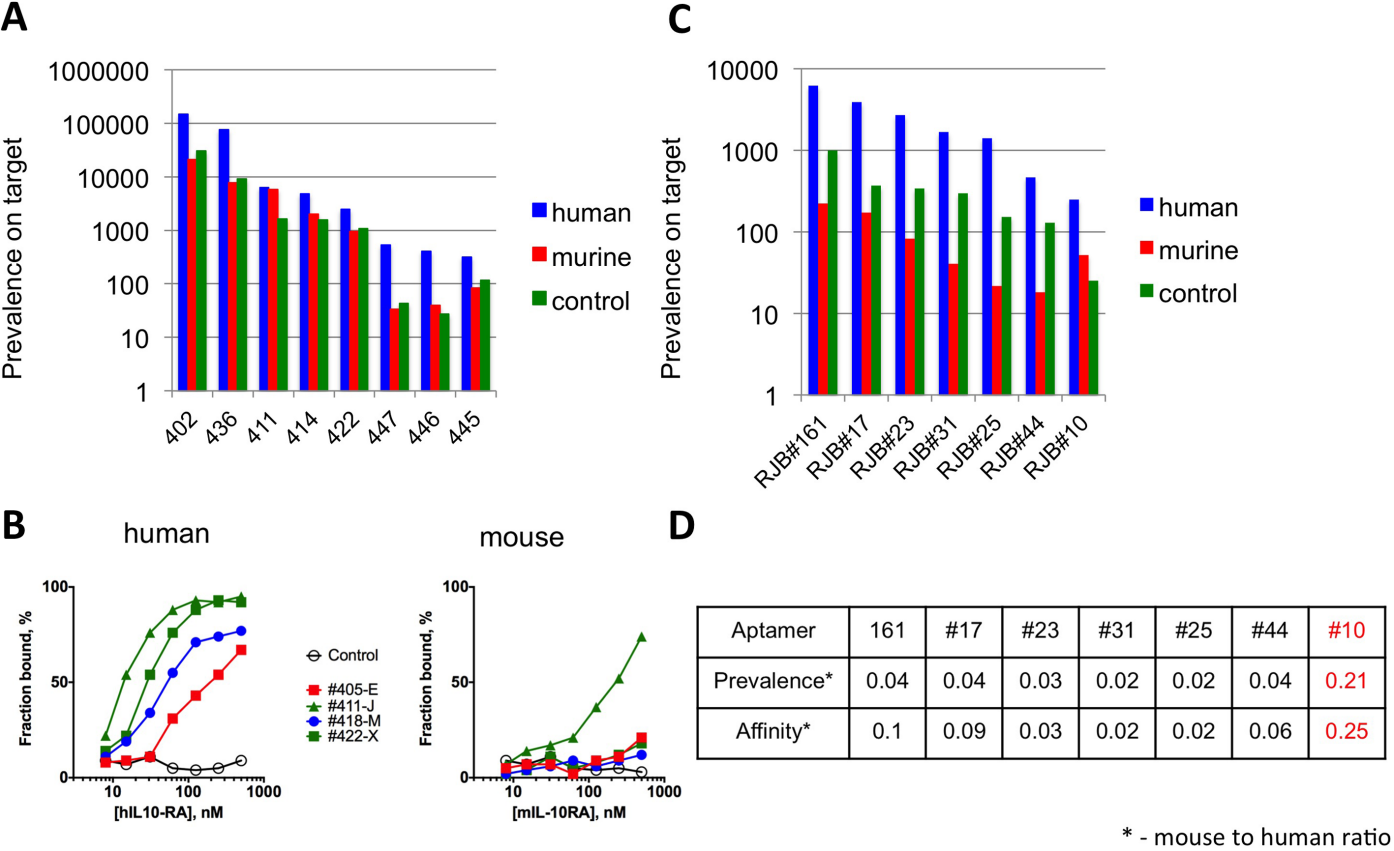
Cross­species aptamers that bind to both murine and human orthologs. (**A**) Cross-species aptamers that bind to both human and murine IL-10RA. After four rounds of selection against the human IL-RA protein, a fifth round of selection was performed against human IL-10RA, murine IL-10RA and hIgG. The prevalences of aptamers identified as high-affinity binders at round 5 are shown for human IL-RA (blue), murine IL-RA (red) and IgG (green). (**B**) Binding of selected aptamers to human or murine IL-10RA protein using a double-filter binding assay (see Materials and Methods section). (**C**) Cross-species aptamers that bind to both human and murine 4-1BB. Same as panel (A), except that the initial five rounds of selection were performed against the human 4-1BB protein. (**D**) Relative prevalence or binding affinity was determined for select 4-1BB aptamers present in round 6 (panel C). Aptamer #10 (red) exhibited high prevalence and affinity to both human and murine 4-1BB proteins.

Comparing the prevalence of the confirmed high-affinity IL-10RA-binding aptamers (Supplementary Table S3) in the mouse and human pools, we found one sequence, #411, that was enriched equally well by both targets (Figure 5A), which was confirmed using a filter-binding assay (Figure 5B). We performed a similar analysis on a library selected against the human 4-1BB-Fc fusion protein and identified one candidate, RBJ#10, that was enriched against both human and murine targets (Figure 5C) and bound to the murine ortholog in a filter-binding assay (Figure 5D and Supplementary Figure S2). Therefore, the analysis of early rounds of HTS-guided SELEX can be used to identify cross-species aptamers capable of binding to both murine and human orthologs.

## DISCUSSION

Aptamers with defined cross-reactivity could be very useful in a variety of biological and medical applications. Such ligands can bind multiple targets, such as human and murine versions of the same protein, while maintaining specificity to those targets alone. Importantly, cross-reactive aptamers can be used both for preclinical studies and as therapeutic ligands in clinical studies, something that is impossible for conventional antibodies and aptamers. Although a number of inventive protocols had been proposed to isolate such ligands, there is currently no established method (5,20). For oligonucleotide ligands, the SELEX procedure can be modified to switch or ‘toggle’ between different targets of interest to generate cross-reactive ligands (14). This approach, however, adds additional unpredictable variables to conventional SELEX and introduces a bias toward lower-affinity cross-reactive species. Here, we use this approach to efficiently isolate aptamers with broad but precise binding properties. Two key elements of the strategy involve expanding the overall variability of the library by optimizing the amplification step and characterizing aptamer binding properties in a highly parallel format.

During *in vitro* selection, a reduction in the overall variability of the library is both expected and necessary, as favorable aptamers become exponentially enriched at the expense of bystander sequences. However, selection pressure is not the only factor that drives the expansion of certain aptamers, as PCR does not amplify all sequences equally, leading to so-called ‘PCR bias’ (21). Here, we demonstrate that traditional PCR has clear biases when amplifying complex mixtures of templates, as sequences with high A:T content are amplified at a higher rate than sequences with high G:C content. Noteworthy, in our hIL-10RA selection at least two G:C out of seven high affinity binders would likely be lost if regular PCR had been used. It is easy to imagine how such preferential amplification could negatively affect selection outcome, and in the worst-case scenario, PCR amplification properties could define the entire resulting pool of candidates isolated during SELEX. The *in vitro* compartmentalization technique allowed us to normalize the amplification rate between different sequences, preserving difficult-to-expand aptamers while effectively capping the over-expansion of sequences with low G:C content that would normally represent false positives. Indeed, ePCR visibly reduced PCR bias, making the binding step of the selection process the most important step with respect to the final results. The net effect of ePCR was overwhelmingly positive, with the variability of the library being preserved to a much greater extent between selection rounds.

The increased sequence diversity preserved by ePCR led to >10-fold increases in the numbers of potential candidates. Whereas regular selection processes can yield 10–50 candidate sequences (or clusters of sequences), >800 individual clusters were identified in the course of this selection. This larger repertoire of potential aptamer candidates is useful for identification of rare ligands with exceptional affinity or binding patterns; however, an abundance of candidates can lead to characterization problems. We confirmed that high prevalence alone, especially at early rounds, is not suggestive of superior binding properties, but rather reflects differences in the amounts of starting template or extensive amplification during early rounds. Instead, we found the expansion rate to be a much more useful indicator of high-affinity aptamers. The most plausible explanation for this observation is that the high affinity of these aptamers enhances their retention in the target-bound pool. This observation is in line with earlier results by Thiel *et al*. (19), demonstrating the great predictive value of aptamer expansion rate.

Because most *in vitro* selection processes are performed on some type of solid support, the enrichment of aptamers binding to the carrier instead of the intended target is quite common. With a notable exception (22), current methods for identifying such sequences are time-consuming *in vitro* binding studies. By contrast, our approach was to incubate the library in question with control targets. Based on enrichment in the control pool, we were able to identify sequences binding the Fc part of the hIL-10RA-Fc fusion protein we were using for selection and to identify sequences that were binding targets in a nonspecific manner.

In a follow-up experiment, the same library was split and exposed to either a murine or a human version of the IL-10 receptor in an attempt to isolate cross-species binding aptamers. Such aptamers were readily identified in the libraries as sequences with similar prevalences following binding to both targets. To generalize this observation, we performed a similar experiment on independently evolved aptamers selected against the human 4-1BB receptor. This selection yielded not only sequences that were highly specific to their original target but also cross-reactive aptamers that were highly enriched in the presence of mouse 4-1BB. Preliminary experiments suggest that this aptamer is able to costimulate polyclonally activated human and murine CD8+ T cells *in vitro*, although the effective concentration of the aptamer for human cells was lower than that for murine cells, likely reflecting a lower affinity for m4-1BB (data not shown). Overall, the enrichment of particular aptamers when incubated with different targets reflects their capacities to bind these targets. Interestingly, the cross-reactivity could be due to presence of a similar epitope in human and murine proteins or due to two different epitopes of these two molecules recognized by two distinct paratopes on the aptamer. Consequently, if two aptamers differs in their ability to cross-react with another target, it could be used as an indication of the differences in the epitope recognition on the original target. Therefore, cross-binding studies could be useful to identify aptamers recognizing different parts of the protein, similar to that approach proposed earlier (23). Although in this study, we use this phenomenon to generate cross-species binding aptamers, in principle, similar approaches could be used to select aptamers against any multiple targets of interest.

The goal of every selection process is to produce a ligand with the highest affinity toward its target. It is becoming quite clear that postselection modifications can improve the specific affinity of most aptamers anywhere from 2- to 10-fold (24); however, the search for such optimized variants is expensive and time-consuming. Here, we demonstrate that HTS information can be used to guide a rational optimization of aptamer sequences to improve their binding properties. We analyzed the prevalence of mutated sequences within high-affinity aptamer clusters and then used this variable to deduce the effects of particular substitutions on binding properties. We found that aptamers with mutations that improved binding tended to be the most prevalent variants within their cluster. Our results indicate that rarely substituted nucleotides correlate with ‘cornerstone’ positions and that mutations at these positions disrupt aptamer binding, which could be useful when optimizing sequences for chemical synthesis to identify positions that should not be altered. The correlation between higher prevalence and enhanced binding helped us to narrow the search for these sequences, although this variable is not a perfect metric, as other factors, such as DNA polymerase errors, likely influence it. The unavoidable downside of this approach is the requirement for a rather high prevalence of the cluster itself within the pool, as well as high confidence in the prevalence data gained by HTS. Of note, most of our samples were HT sequenced just once, and therefore, we could not measure the statistical power of our observations. In a separate paper, we discuss another way to address this limitation by analyzing the expansion rate of single-nucleotide polymorphism (SNP)-bearing variants instead of prevalence alone [Hoinka *et al*., manuscript in press]. While more mechanistic and comparative studies should be performed, we believe that substitution pattern analysis could be used to guide the selection of aptamers with superior binding affinity.

## SUPPLEMENTARY DATA

Supplementary Data are available at NAR Online.

SUPPLEMENTARY DATA
