# Supplementary material for: Identifying high-affinity aptamer ligands with defined cross-reactivity using high-throughput guided systematic evolution of ligands by exponential enrichment
Source: Nucleic Acids Res. 2015 May 24;43(12):e82. doi: 10.1093/nar/gkv534 (PMC4499151; doi:10.1093/nar/gkv534)
Supplement: SUPPLEMENTARY DATA [file supp_43_12_e82__index.html]

Identifying high-affinity aptamer ligands with defined cross-reactivity using high-throughput guided systematic evolution of ligands by exponential enrichment — Identifying high-affinity aptamer ligands with defined cross-reactivity using high-throughput guided systematic evolution of ligands by exponential enrichment — Identifying high-affinity aptamer ligands with defined cross-reactivity using high-throughput guided systematic evolution of ligands by exponential enrichment — SUPPLEMENTARY DATA 

# Identifying high-affinity aptamer ligands with defined cross-reactivity using high-throughput guided systematic evolution of ligands by exponential enrichment

## SUPPLEMENTARY DATA

- SUPPLEMENTARY DATA
