# Supplementary material for: Identifying high-affinity aptamer ligands with defined cross-reactivity using high-throughput guided systematic evolution of ligands by exponential enrichment
Source: Nucleic Acids Res. 2015 May 24;43(12):e82. doi: 10.1093/nar/gkv534 (PMC4499151; doi:10.1093/nar/gkv534)
Supplement: SUPPLEMENTARY DATA [file supp_gkv534_nar-00453-met-n-2015-File010.pdf]

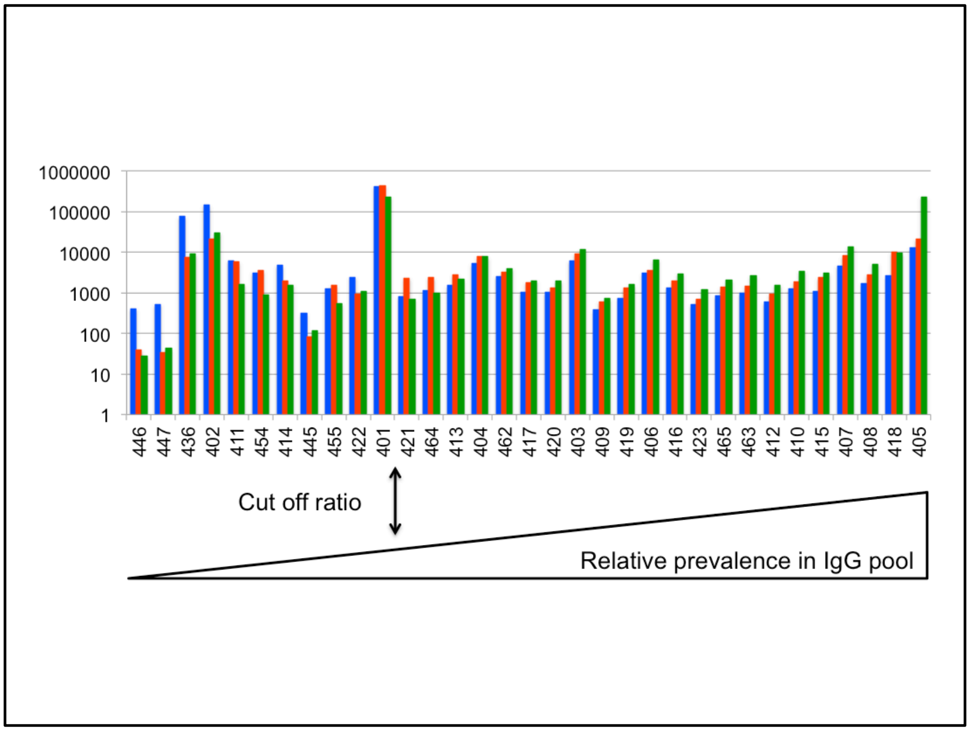

Supplementary figure 1. *Nonspecific binders enriched on positively charged mIL-10RA protein.*
Prevalences of aptamers from major clusters at round 5 in the pools isolated from human IL10RA protein (blue), murine IL-10RA protein (red), or IgG protein (green) were determined by high-throughput sequencing, as described in the Methods. Sequences were ranked by a specificity index that was determined as the hIL-10RA prevalence divided by the hIgG prevalence. The threshold was set at a hIL-10RA to hIgG ratio greater than 1.

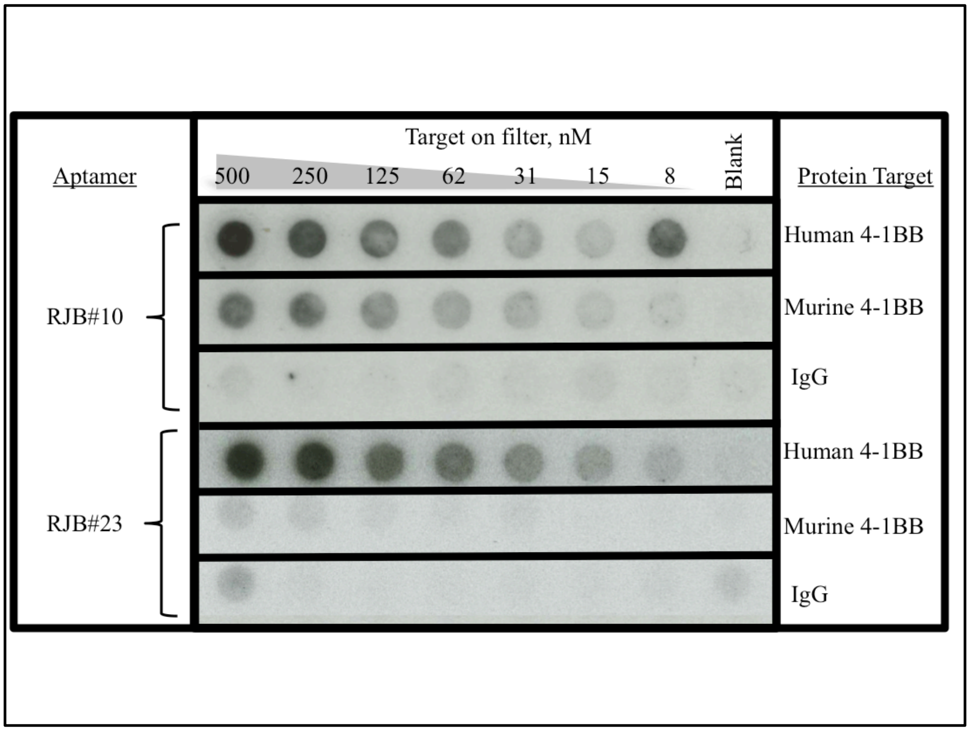

Supplementary figure 2. *Correlation between prevalences and binding patterns of h4-1BB aptamers.*

Radiolabeled aptamers with high (RJB#10) and low (RJB#23) prevalences in the murine 4-1BB pool were incubated with decreasing concentrations of human 4-1bb protein (lanes 1 and 4), murine 4-1BB protein (lanes 2 and 5) and control (lanes 3 and 6). Binding properties were visualized by retention of the protein:RNA complex on a nitrocellulose filter.

Supplementary table S1. *Conditions of hIL10-RA aptamer in vitro selection*

Round	Amount of protein, ug	Amount of RNA, nM	NaCl concentration
1.	25	2	150
2.	20	1.5	150
3.	16	1	150
4.	12	1	150
5.	10	1	150

Supplementary table S2. *Amplification of round 2 hIL-10RA candidate aptamers via emulsion or open PCR.*

N40 sequence	GC content*	Prevalence at round 2	Prevalence at round 3		Rate of amplification R2->R3	
			ePCR	oPCR	ePCR	oPCR
CATGACACATAGTAGAACGCGCAAAAATTATTGAAGTAAA	0.325	96	109	1033	1.14	10.76
ACTATAACGCGTCAAAGTGCTTATCGAACACTATTTGTAA	0.35	615	807	463	1.31	0.75
TAGACGAGCACTTCTCTCAGTCGCATTCATTATTTAAATT	0.35	435	565	699	1.30	1.61
CATGACACATAGAATATAACGCGCAAATCACAGTCATATG	0.375	152	239	1052	1.57	6.92
TGCAATGAGGACTTCTCTCAGTCTAACACAATGTTGTTTA	0.375	136	150	61	1.10	0.45
TATAGAGAACTTCTCTCAGTCGAAGCCAAAGAGCATTAAT	0.375	135	115	75	0.85	0.56
ATGAGGACTTCTCTCAGTCGTGACCATTAAATAAGAGAAA	0.375	117	128	121	1.09	1.03
AGCAAAGTCGTCAGCAATATAACACTCGATTCTTATTGGA	0.375	84	140	280	1.67	3.33
CCACTTCAATTTTCACGTCAAAATACAGACGTGACTGAAA	0.375	37	72	36	1.95	0.97
ACGATCACTTCTCTCAGTCGGACTAATATTACGGTTAGAA	0.4	142	233	79	1.64	0.56
CATGACACATCGGATCAATAAGATCGAAAACGCGCAAGAA	0.425	59	122	61	2.07	1.03
TGTCGAGTACTTTTCTCAGTCTATTCCACAGTAGTGGAGA	0.425	58	92	17	1.59	0.29
CAAAGCGAGTTACGAAGAACTTGAATCTCAAGGTGCTCTT	0.425	39	36	17	0.92	0.44
ATCGACAGCTCTCAGTCCATTCGAGGAATGTTCATCGATA	0.45	145	186	32	1.28	0.22
TGCCCTGCTTCCCCAGTCTTGTCTCACAAGACTAATTTTA	0.45	142	207	140	1.46	0.99
TTGAGGACTTCTCTCAGTCAAAAGGACATTAGGGACCTGA	0.45	110	135	25	1.23	0.23
GTTACCTCTGCATAGGAGGCCTAGGAATAATTTAATGCGC	0.45	87	134	181	1.54	2.08
TATGTTTATCAGCGTACTTCGGGTGCTCCTGTATTGGAAC	0.45	60	84	85	1.40	1.42
TGAGAACTTCTCTCAGTCGGTGGGAGAGTACATCCTAACA	0.475	653	928	372	1.42	0.57
AGCCATGACGATGTCGTTACGTAGATGCAGAGACTCCTAA	0.475	90	192	125	2.13	1.39
TTCTCAGTCAATAACTAGTCGGATCGTGGTCCAAGAGCAG	0.475	44	165	25	3.75	0.57
ATTGAGCACTTCTCGCAGTCGCACTGTACAATAGCGTATG	0.475	34	53	15	1.56	0.44
TAGTCTCCCAAGACAGCGACTGACGAAAGCTCTGGTACTA	0.5	94	198	150	2.11	1.60
ATCATATTCTCGCGAGCCGGAACTCAACTTAGAGGTGCTC	0.5	67	83	22	1.24	0.33
TAAGGTCGAACGGAACTGGTTTAGTACCAGGTGCTCTGGT	0.5	42	33	34	0.79	0.81
TCCAGCGTAGGAACTCTAATGCATGACATACGAGGTGCTC	0.5	40	53	9	1.33	0.23
CACGCGAGGACTTCTCTCAGTCGGTACCTAACTAAGAGTT	0.5	32	46	30	1.44	0.94
CCCCTTCCAGCGATTACGATCATTGACTCTCAGTCCTGTG	0.525	320	445	68	1.39	0.21

AGAGTTAAGACCGTCGGTGCTCCATGTTCCCTCCATGGAA	0.525	128	162	32	1.27	0.25
ACACGACGAGTACAGCTCTCAGTCGAGCGTATTAGCGGAT	0.525	80	131	39	1.64	0.49
GACTCAGCAGCGCGAGAAAGACGTGGTTAGCCTCAATATG	0.525	74	128	88	1.73	1.19
TAACACTCGATTCTCCTAGCCCGCTAGAAATTCCCCTCCC	0.525	66	133	546	2.02	8.27
TGCTCCGCTGTGACGCGGAACCATCTAGACAATGCATACA	0.525	62	72	0	1.16	0.00
TCACAGTCCCGGTGCCGCACTAAAACCCATTGTTGTGCGA	0.55	1056	1372	428	1.30	0.41
TCCAAGGAACTAGGCAGGGCGTAAACCCGAATAGGTGCTC	0.55	64	47	26	0.73	0.41
CCCACGCATCACGCCGTGGTGCGATTGACACAATTGCAAT	0.55	35	18	0	0.51	0.00
CAACGAACTCACAAACCTTTCATAAGCCGCCCCTCGCGCA	0.55	32	59	11	1.84	0.34
CCCCCGCATCACGCCGTGGTGCGATTGACACAATTGCAAT	0.575	2572	3027	766	1.18	0.30
CTCAGCGCGTAGTCGTGTGCGAAACTGCCTTTCCATGGTC	0.575	64	100	10	1.56	0.16
AATAGCTCAGCCGGTCCGGAACTGGCAAAGTCAGGTGCTC	0.575	57	20	0	0.35	0.00
CCCCCGCATCTCGCCGTGGTGCGATTGACACAATTGCAAT	0.575	55	74	0	1.35	0.00
CTGCGTCCGGAACCTGATCCTTGATCAGGGTGCTCTAGGA	0.575	42	59	0	1.40	0.00
AATCGCTCAGCCGGTCCGGAACTGGCAAAGTCAGGTGCTC	0.6	2171	2335	217	1.08	0.10
GTACAGCGACTCATCGGGTGCTCCCGGTTGCCAAGGGAAC	0.625	68	55	20	0.81	0.29
GAGAGAACTTCTCTCAGTCGGGACCCGCCAACCGGCAACC	0.625	61	100	72	1.64	1.18
CTTATAGGGAACCGGCAGGAAGCCGGGTGCTCCCTAGACG	0.625	40	55	19	1.38	0.48
ACTCGCTCAGCCGGTCCGGAACTGGCAAAGTCAGGTGCTC	0.625	36	25	0	0.69	0.00
TTCCCCCCGACCCGCACGTCCATGGTCCGTTACACGTGTG	0.65	72	116	14	1.61	0.19
GACACGCGAGCCTGTCGGGAACTCTTGTGCGAGGTGCTCC	0.65	33	44	48	1.33	1.45
TCCGCCGGAACCGGCCTCCGAATAGGAGTGGCGGGTGCTC	0.7	37	14	0	0.38	0.00

Supplementary table S3. Prevalence, expansion rate and binding properties of aptamers isolated in hIL-10RA selection

	Round	2	3	4		5			4->5 expansion rate	Affinity to			
ID	Sequence	hIL-10RA	hIL-10RA	hIL-10RA	hIgG (pH 7.5)	hIL-10RA	mIL-10RA	IgG (pH 6.8)		hIL-10RA Kd, nM	mIL-10RA Kd, nM	hTrkB Kd, nM	hIgG Kd, nM
401	CCCCCGCATCACGCCGTGGTGCGATTGACACAATTGCAAT	620.5	1390.1	103513.1	126.5	421605.7	430594.5	232234.7	4.07	25	65		>500
402	TCACAGTCCCGGTGCCGCACTAAAACCCATTGTTGTGCGA	254.8	630.1	37630.2	91.4	149129.3	21152.9	30288.8	3.96	120			
403	TGAGAACTTCTCTCAGTCGGTGGGAGAGTACATCCTAACA	157.5	426.2	23423.0	38.7	6081.4	9251.0	11878.6	0.26	>500			
404	ACTATAACGCGTCAAAGTGCTTATCGAACACTATTTGTAA	148.4	370.6	20888.5	55.4	5248.7	7858.0	7808.1	0.25	50			
405	AATCGCTCAGCCGGTCCGGAACTGGCAAAGTCAGGTGCTC	523.8	1072.3	19281.9	123202.2	13084.1	21020.4	235665.7	0.68	60			120
406	TAGACGAGCACTTCTCTCAGTCGCATTCATTATTTAAATT	104.9	259.5	16011.0	21.3	3058.3	3670.2	6551.4	0.19	>500			
407	CCCCTTCCAGCGATTACGATCATTGACTCTCAGTCCTGTG	77.2	204.4	11789.2	22.6	4678.9	8150.4	13777.0	0.40	>500			
408	ATCGACAGCTCTCAGTCCATTCGAGGAATGTTCATCGATA	35.0	85.4	5636.9	12.3	1738.1	2765.0	5184.1	0.31	>120			
409	ACACGACGAGTACAGCTCTCAGTCGAGCGTATTAGCGGAT	19.3	60.2	3479.5	14.2	376.5	608.0	740.6	0.11	>500			
410	ACGATCACTTCTCTCAGTCGGACTAATATTACGGTTAGAA	34.3	107.0	5980.7	9.3	1272.2	1896.9	3411.8	0.21	>500			
**411**	**AGCCATGACGATGTCGTTACGTAGATGCAGAGACTCCTAA**	**21.7**	**88.2**	**3960.6**	**33.3**	**6311.1**	**5817.8**	**1648.4**	**1.59**	**18**	**50**	**>500**	**>500**
412	TTCCCCCCGACCCGCACGTCCATGGTCCGTTACACGTGTG	17.4	53.3	2803.4	8.8	591.1	952.6	1570.6	0.21	>120			
413	CATGACACATAGAATATAACGCGCAAATCACAGTCATATG	36.7	109.8	5195.9	41.9	1520.6	2821.5	2148.4	0.29	40			
414	AGCAAAGTCGTCAGCAATATAACACTCGATTCTTATTGGA	20.3	64.3	3191.9	23.0	4771.7	2005.3	1581.7	1.49	80			
415	TATAGAGAACTTCTCTCAGTCGAAGCCAAAGAGCATTAAT	32.6	52.8	5264.0	6.4	1097.9	2366.8	3032.6	0.21	>500			
416	TGCAATGAGGACTTCTCTCAGTCTAACACAATGTTGTTTA	32.8	68.9	3954.4	10.1	1364.6	1964.2	2957.1	0.35	>500			
417	TAGTCTCCCAAGACAGCGACTGACGAAAGCTCTGGTACTA	22.7	90.9	3248.1	27.5	1045.9	1827.9	2025.9	0.32	>500			
418	GTTACCTCTGCATAGGAGGCCTAGGAATAATTTAATGCGC	21.0	61.5	2777.4	118.7	2604.0	9982.9	9593.7	0.94	35		80	>500
419	TGCCCTGCTTCCCCAGTCTTGTCTCACAAGACTAATTTTA	34.3	95.1	4951.4	8.1	750.0	1320.0	1586.7	0.15	>500			
420	TGTCGAGTACTTTTCTCAGTCTATTCCACAGTAGTGGAGA	14.0	42.3	2125.7	6.9	1018.8	1329.2	1974.3	0.48	80			
421	TTCTCAGTCAATAACTAGTCGGATCGTGGTCCAAGAGCAG	10.6	75.8	1921.8	40.9	817.5	2334.3	700.3	0.43	20	80	>500	
422	GACTCAGCAGCGCGAGAAAGACGTGGTTAGCCTCAATATG	17.9	58.8	2605.7	22.3	2466.9	948.5	1089.5	0.95	20		>250	
423	ATGAGGACTTCTCTCAGTCGTGACCATTAAATAAGAGAAA	28.2	58.8	3735.4	5.9	512.3	692.5	1212.8	0.14	>500			
436	TAACACTCGATTCTCCTAGCCCGCTAGAAATTCCCCTCCC	15.9	61.1	2512.6	32.4	76373.5	7733.7	9134.3	30.40	65			
445	CCGCTAACACTCGATTCTGCGGAAATGCCCCCCTGAACCC	0.2	1.4	88.9	2.2	317.7	84.3	116.4	3.57	>120			
**446**	**AAAGACCGTTTTTTAAAACGCTCAATATACACGACATAAA**	**0.7**	**3.7**	**88.9**	**0.2**	**409.0**	**39.4**	**27.2**	**4.60**	**10**	**>500**	**>500**	**>500**
447	ACAGACCAAGTGTTCAGAAAAACAGTTGCTCAATATACAT	0.5	2.3	110.3	1.7	530.1	33.9	42.8	4.81	25			
**454**	**TGAATCTCGCGCTCGTTGGTACCCTTAAAATAAAGGCATA**	**4.3**	**36.7**	**741.7**	**139.5**	**3147.4**	**3581.6**	**892.8**	**4.24**	**8**	**120**		**>500**
**455**	**CGTGACTCGACTCAGGTTTTGCACGGCCTCAGGGTAGCAC**	**5.1**	**25.3**	**811.9**	**640.6**	**1262.7**	**1537.2**	**555.9**	**1.56**	**4**	**120**		**>500**
**462**	**CTCAGCGCGTAGTCGTGTGCGAAACTGCCTTTCCATGGTC**	**15.4**	**45.9**	**2466.3**	**15.7**	**2599.4**	**3263.4**	**3978.8**	**1.05**	**2**	**60**		**>500**
**463**	**GAGAGAACTTCTCTCAGTCGGGACCCGCCAACCGGCAACC**	**14.7**	**45.9**	**2579.7**	**5.6**	**1004.2**	**1485.6**	**2646.5**	**0.39**	**12**			
**464**	**CATGACACATAGTAGAACGCGCAAAAATTATTGAAGTAAA**	**23.2**	**50.1**	**3129.0**	**15.0**	**1131.3**	**2377.0**	**1003.1**	**0.36**	**4**	**80**		**>500**
465	TTGAGGACTTCTCTCAGTCAAAAGGACATTAGGGACCTGA	26.5	62.0	3832.1	5.6	838.2	1388.0	2057.6	0.22	25			

*-sequences with high affinity to hIL-10RA (respective Kds below 20 nM) boldened in the list.
** - sequences unable to bind hIL-10RA target highlighted grey, sequence cross-reacting with a carrier protein (hIgG) highlighted red, sequence cross-reactive with murine IL-10RA highlighted green and sequence with similar abundance at hIL-10RA and hIgG and therefore considered to have significant nonspecific component in their binding to hIL-10RA highlighted blue

Supplementary table S4. *Conditions of h4-1BB aptamer in vitro selection*

Round	Amount of protein, ug	Amount of RNA, nM	NaCl concentration, nM
1.	10	1,5	50
2.	10	1	75
3.	8	1	100
4.	6	1	125
5.	5	0,75	150

Supplementary table S5. Aptamers isolated in h4-1BB selection and their properties.

ID	N25 sequence	Round 4 H4-1BB	Round 5 H4-1BB	Round 5 m4-1BB	Round 5 hIgG	R4->5 expansion rate	Mouse to human prevalence ratio	Mouse to human affinity ratio	Affinity to human 4-1BB*, nM	Affinity to murine 4-1BB, nM	Affinity to human IgG, nM
RJB#1	TCGAGTCCCTAAGTTTTCTCGCATATGTGC	517	8664	93	563	16.8	0.01				
RJB#15	TCGAGTCCCTATGTTGTCTTTCGCTCTAGC	11	177	3	10	16.3	0.02				
**RJB#17**	**TGCTCCTACTGTGGAACCATCGTTCTTGTG**	**241**	**3917**	**173**	**369**	**16.3**	**0.04**	**<0.09**	**35**	**>500**	**>1000**
RJB#38	TTGAGTCCCTATGTAGTCTTGCAATGTAGC	74	1148	5	73	15.5	0.00				
RJB#54	TGCTCATTTTGAACCATCGTTTCTCGGCGC	171	2498	63	265	14.6	0.03		200		
RJB#19	TGCTCTATTCCCACGTCTTCATTGTCACCG	82	1154	3	104	14.1	0.00		>500		
RJB#16	TGCTCATGTAATTTGAACCATCGTTCTGCC	31	414	13	30	13.5	0.03				
**RJB#23**	**TGCTCACAAACGTGAACCATCGTCTTGTGC**	**215**	**2708**	**83**	**340**	**12.6**	**0.03**	**<0.03**	**30**	**>1000**	**>1000**
**RJB#25**	**TGCTCAATTAAGAACCATCGCTCTCCGGTG**	**119**	**1410**	**22**	**153**	**11.8**	**0.02**	**<0.02**	**25**	**>1000**	**>1000**
RJB#18	TGCTCCATGTGGAACCATTGTTCTTGTCCC	37	430	38	54	11.8	0.09				
RJB#35	TCGAGTCTCTATGTTGTCTAGCCTTACGGT	33	362	6	22	11.1	0.02				
**RJB#161**	**TGCTCGTTTCGAACCATCGTAACTCCTGTG**	**576**	**6188**	**223**	**994**	**10.7**	**0.04**	**0.13**	**20**	**150**	**>1000**
RJB#108	AGGTGCTCATTAATGAACTCCGCTTGTCTG	19	186	9	30	9.9	0.05				
RJB#12	TCGAGTCCCATACGTAGTCTTGCTTAATGC	161	1592	8	138	9.9	0.01				
RJB#67	TGTTCGTCCACATTCGAAACTCGTTGTCTG	51	502	57	215	9.8	0.11				
RJB#8	TCGTCCTGACGTTTGAGACTTGTCGTTCTG	59	534	464	123	9.0	0.87				
**RJB#31**	**TGCTCATCTTGAACGCATTGTTTCTCCGTG**	**188**	**1673**	**41**	**297**	**8.9**	**0.02**	**<0.02**	**20**	**>1000**	**>500**
RJB#47	TGCTCCTAAATCGGAACCATCGTCTTCCGG	67	583	12	54	8.7	0.02				
RJB#45	TGCTCATTCTTGAACGCTCGTTATCTCCTG	37	324	8	57	8.7	0.03		60		
RJB#44	TGCTTCATAGAAACGCGCGATCTTCCTGTG	56	468	18	130	8.3	0.04	<0.06	60	>1000	>1000
RJB#2	TCCCATCTTCCCGATTCTCAACATCGTGTG	878	6868	892	2028	7.8	0.13				

**RJB#10**	**TGCTCACATACGTGAACCATCGTCAATGGC**	**33**	**250**	**52**	**25**	**7.7**	**0.21**	**0.25**	**30**	**120**	**>1000**
RJB#102	TACGTAATCGTCACTGAAGCAGCGTTTGGC	77	590	36	142	7.7	0.06				
RJB#74	TGCTCCTCATTGGAACGCTTCGTTCGTCTG	224	1678	212	413	7.5	0.13		200		
RJB#93	TGCTCAACATTGAACCATCGTTCCAGTGGC	165	1162	26	171	7.1	0.02		120		
RJB#59	TGCTCCATGAGTGGAACGCATTTTGTCTGG	149	1032	57	112	6.9	0.05		40		
RJB#3	CGTCCGTTTGGGTAACCATATATTTGGCCC	176	1133	8	181	6.5	0.01				
RJB#127	TGCTCATAATGAACCATTGTCCTCGTTGGG	20	126	2	22	6.4	0.01		>500		
RJB#75	TAACGCATCACTGAAGTGAGCCTTCAGCGA	40	254	0	17	6.3	0.00				
RJB#7	ACTATTGCGTAGCACACGCTTTCTTTGTGC	54	331	3	60	6.1	0.01				
RJB#231	TGCTCATACGAACGTATCGTCCATTTGCCG	105	629	23	170	6.0	0.04				
RJB#6	TCACGCGCTCTCTTCGTGATTATTCCTCCC	48	273	5	33	5.6	0.02				
RJB#4	GCGCTTACGTTATGCCTCTCATAGATCGTG	131	701	41	131	5.3	0.06				
RJB#294	GTCCTACGTGATGAAACTTGTCGTTTGGTG	142	708	378	431	5.0	0.53				
RJB#13	CGTCTCGTCAATGCCCTACGTTTGTCTGTG	187	793	95	269	4.3	0.12		>1000	>1000	>1000
RJB#14	TTCGTCCTGTTACAAACCTCGCTCGTCTGG	88	270	79	126	3.1	0.29				
RJB#9	TAACACCATCTTCCCGATTCTCAAACTGGC	302	921	59	405	3.0	0.06				

*-sequences with high affinity to h4-1BB boldened in the list

Supplementary table S6. Sequences belonging to cluster #411-J and their prevalence at round 5 of selection

ID	Sequence	Round 5 prevalence	Murine R5 prevalence
411	AGCCATGACGATGTCGTTACGTAGATGCAGAGACTCCTAA	6311	5818
A1G	GGCCATGACGATGTCGTTACGTAGATGCAGAGACTCCTAA	47	44
G30T	AGCCATGACGATGTCGTTACGTAGATGCATAGACTCCTAA	46	31
C4G	AGCGATGACGATGTCGTTACGTAGATGCAGAGACTCCTAA	35	24
G32T	AGCCATGACGATGTCGTTACGTAGATGCAGATACTCCTAA	29	20
A5C	AGCCCTGACGATGTCGTTACGTAGATGCAGAGACTCCTAA	25	34
T12C	AGCCATGACGACGTCGTTACGTAGATGCAGAGACTCCTAA	25	18
G7T	AGCCATTACGATGTCGTTACGTAGATGCAGAGACTCCTAA	25	18
G10A	AGCCATGACAATGTCGTTACGTAGATGCAGAGACTCCTAA	22	25
T6C	AGCCACGACGATGTCGTTACGTAGATGCAGAGACTCCTAA	21	16
C4A	AGCAATGACGATGTCGTTACGTAGATGCAGAGACTCCTAA	21	17
G2A	AACCATGACGATGTCGTTACGTAGATGCAGAGACTCCTAA	21	17
C9T	AGCCATGATGATGTCGTTACGTAGATGCAGAGACTCCTAA	20	22
G7A	AGCCATAACGATGTCGTTACGTAGATGCAGAGACTCCTAA	20	17
G27T	AGCCATGACGATGTCGTTACGTAGATTCAGAGACTCCTAA	19	15
G13T	AGCCATGACGATTTCGTTACGTAGATGCAGAGACTCCTAA	19	16
A8G	AGCCATGGCGATGTCGTTACGTAGATGCAGAGACTCCTAA	17	15
C28T	AGCCATGACGATGTCGTTACGTAGATGTAGAGACTCCTAA	17	15
G10C	AGCCATGACCATGTCGTTACGTAGATGCAGAGACTCCTAA	14	11
T17C	AGCCATGACGATGTCGCTACGTAGATGCAGAGACTCCTAA	13	16
T26C	AGCCATGACGATGTCGTTACGTAGACGCAGAGACTCCTAA	12	8
T38C	AGCCATGACGATGTCGTTACGTAGATGCAGAGACTCCCAA	11	9
T14C	AGCCATGACGATGCCGTTACGTAGATGCAGAGACTCCTAA	11	11
A8C	AGCCATGCCGATGTCGTTACGTAGATGCAGAGACTCCTAA	11	9
T35C	AGCCATGACGATGTCGTTACGTAGATGCAGAGACCCCTAA	10	12
A29T	AGCCATGACGATGTCGTTACGTAGATGCTGAGACTCCTAA	10	10
G10T	AGCCATGACTATGTCGTTACGTAGATGCAGAGACTCCTAA	10	11
T18C	AGCCATGACGATGTCGTCACGTAGATGCAGAGACTCCTAA	10	6
T22C	AGCCATGACGATGTCGTTACGCAGATGCAGAGACTCCTAA	10	10
C15T	AGCCATGACGATGTTGTTACGTAGATGCAGAGACTCCTAA	10	7
A29G	AGCCATGACGATGTCGTTACGTAGATGCGGAGACTCCTAA	9	10
G30A	AGCCATGACGATGTCGTTACGTAGATGCAAAGACTCCTAA	9	8
A31G	AGCCATGACGATGTCGTTACGTAGATGCAGGGACTCCTAA	8	8
C36T	AGCCATGACGATGTCGTTACGTAGATGCAGAGACTTCTAA	8	6
C4T	AGCTATGACGATGTCGTTACGTAGATGCAGAGACTCCTAA	8	7
C20T	AGCCATGACGATGTCGTTATGTAGATGCAGAGACTCCTAA	8	8
C37T	AGCCATGACGATGTCGTTACGTAGATGCAGAGACTCTTAA	8	6
A5G	AGCCGTGACGATGTCGTTACGTAGATGCAGAGACTCCTAA	7	7

A33C	AGCCATGACGATGTCGTTACGTAGATGCAGAGCCTCCTAA	7	4
A33G	AGCCATGACGATGTCGTTACGTAGATGCAGAGGCTCCTAA	7	8
G21A	AGCCATGACGATGTCGTTACATAGATGCAGAGACTCCTAA	7	4
G24T	AGCCATGACGATGTCGTTACGTATATGCAGAGACTCCTAA	7	8
G16T	AGCCATGACGATGTCTTTACGTAGATGCAGAGACTCCTAA	7	7
C3T	AGTCATGACGATGTCGTTACGTAGATGCAGAGACTCCTAA	6	6
T12G	AGCCATGACGAGGTCGTTACGTAGATGCAGAGACTCCTAA	6	4
A8T	AGCCATGTCGATGTCGTTACGTAGATGCAGAGACTCCTAA	6	4
A40G	AGCCATGACGATGTCGTTACGTAGATGCAGAGACTCCTAG	6	6
C3A	AGACATGACGATGTCGTTACGTAGATGCAGAGACTCCTAA	6	5
C34T	AGCCATGACGATGTCGTTACGTAGATGCAGAGATTCCTAA	6	4
G27A	AGCCATGACGATGTCGTTACGTAGATACAGAGACTCCTAA	6	4
G21T	AGCCATGACGATGTCGTTACTTAGATGCAGAGACTCCTAA	6	7
A39G	AGCCATGACGATGTCGTTACGTAGATGCAGAGACTCCTGA	5	7
A25G	AGCCATGACGATGTCGTTACGTAGGTGCAGAGACTCCTAA	5	4
A11T	AGCCATGACGTTGTCGTTACGTAGATGCAGAGACTCCTAA	5	3
G2T	ATCCATGACGATGTCGTTACGTAGATGCAGAGACTCCTAA	5	9
C9A	AGCCATGAAGATGTCGTTACGTAGATGCAGAGACTCCTAA	5	4
A23G	AGCCATGACGATGTCGTTACGTGGATGCAGAGACTCCTAA	5	4
G24A	AGCCATGACGATGTCGTTACGTAAATGCAGAGACTCCTAA	5	2
A11G	AGCCATGACGGTGTCGTTACGTAGATGCAGAGACTCCTAA	4	4
C36A	AGCCATGACGATGTCGTTACGTAGATGCAGAGACTACTAA	4	2
A1C	CGCCATGACGATGTCGTTACGTAGATGCAGAGACTCCTAA	4	2
G13A	AGCCATGACGATATCGTTACGTAGATGCAGAGACTCCTAA	4	4
A33T	AGCCATGACGATGTCGTTACGTAGATGCAGAGTCTCCTAA	4	3
T6G	AGCCAGGACGATGTCGTTACGTAGATGCAGAGACTCCTAA	4	4
G32A	AGCCATGACGATGTCGTTACGTAGATGCAGAAACTCCTAA	4	4
A31T	AGCCATGACGATGTCGTTACGTAGATGCAGTGACTCCTAA	4	3
G16A	AGCCATGACGATGTCATTACGTAGATGCAGAGACTCCTAA	3	3
A5T	AGCCTTGACGATGTCGTTACGTAGATGCAGAGACTCCTAA	3	2
A19G	AGCCATGACGATGTCGTTGCGTAGATGCAGAGACTCCTAA	3	3
G7C	AGCCATCACGATGTCGTTACGTAGATGCAGAGACTCCTAA	3	3
T12A	AGCCATGACGAAGTCGTTACGTAGATGCAGAGACTCCTAA	3	2
G2C	ACCCATGACGATGTCGTTACGTAGATGCAGAGACTCCTAA	3	4
G30C	AGCCATGACGATGTCGTTACGTAGATGCACAGACTCCTAA	3	1
T14A	AGCCATGACGATGACGTTACGTAGATGCAGAGACTCCTAA	3	2
A40T	AGCCATGACGATGTCGTTACGTAGATGCAGAGACTCCTAT	3	2
T38A	AGCCATGACGATGTCGTTACGTAGATGCAGAGACTCCAAA	3	2
T14G	AGCCATGACGATGGCGTTACGTAGATGCAGAGACTCCTAA	2	3
A19T	AGCCATGACGATGTCGTTTCGTAGATGCAGAGACTCCTAA	2	2
A40C	AGCCATGACGATGTCGTTACGTAGATGCAGAGACTCCTAC	2	1
